# A universal synthetic route to carbon nanotube/transition metal oxide nano-composites for lithium ion batteries and electrochemical capacitors

**DOI:** 10.1038/srep37752

**Published:** 2016-11-25

**Authors:** Han Zhou, Lusi Zhang, Dongyang Zhang, Shuangqiang Chen, Paul R. Coxon, Xiong He, Mike Coto, Hyun-Kyung Kim, Kai Xi, Shujiang Ding

**Affiliations:** 1 Department of Applied Chemistry, School of Science, MOE Key Laboratory for Nonequilibrium Synthesis and Modulation of Condensed Matter, State Key Laboratory for Mechanical Behaviour of Materials, Xi’an Jiaotong University, Xi’an China; 2 Centre for Clean Energy Technology, School of Mathematical and Physical Sciences, University of Technology Sydney, 15 Broadway, Sydney, New South Wales, 2007, Australia; 3 Department of Materials Science and Metallurgy, University of Cambridge, Cambridge CB3 0FS, United Kingdom

## Abstract

We report a simple synthetic approach to coaxially grow transition metal oxide (TMO) nanostructures on carbon nanotubes (CNT) with ready control of phase and morphology. A thin (~4 nm) sulfonated-polystyrene (SPS) pre-coating is essential for the deposition of transition metal based materials. This layer has abundant sulfonic groups (−SO_3_^−^) that can effectively attract Ni^2+^, Co^2+^, Zn^2+^ ions through electrostatic interaction and induce them via hydrolysis, dehydration and recrystallization to form coaxial (NiO, Co_3_O_4_, NiCoO_2_ and ZnCo_2_O_4_) shells and a nanosheet-like morphology around CNT. These structures possess a large active surface and enhanced structural robustness when used as electrode materials for lithium-ion batteries (LIBs) and electrochemical capacitors (ECs). As electrodes for LIBs, the ZnCo_2_O_4_*@*CNT material shows extremely stable cycling performance with a discharge capacity of 1068 mAh g^−1^ after 100 cycles at a current density of 400 mAg^−1^. For EC applications, the NiCoO_2_*@*CNT exhibits a high capacitance of 1360 Fg^−1^ at current densities of 10 Ag^−1^ after 3000 cycles and an overall capacitance loss of only 1.4%. These results demonstrate the potential of such hybrid materials meeting the crucial requirements of cycling stability and high rate capability for energy conversion and storage devices.

The ability to simply design and engineer new materials with tailor-made functionality is an important goal in materials science, and new classes of designer nanoscale materials will likely play a decisive role in the development of future technologies. Carbon nanotubes (CNTs) have received great interest following their discovery by Iijima in 1991^1^, owing to their excellent mechanical, thermal, and electrical properties^2^,^3^,^4^,^5^. In order to harness the exceptional properties of CNTs, one strategy is to exploit their applications and composite structures with other functional materials, such as polymers, metals and metal oxides/sulfides/nitrides/phosphides^2^,^3^,^6^,^7^,^8^,^9^,^10^,^11^. Within these hybrid materials, CNTs typically serve as the support while the functional components fill the cavities and/or are coated on the outer surface^2^,^3^,^4^,^6^,^9^,^10^,^12^,^13^,^14^,^15^,^16^.

Transition metal oxides (TMOs) as promising functional materials have been widely studied for catalysis applications, gas sensors, energy conversion and storage devices due to their abundance, environmental friendliness and specific physical/chemical properties^4^,^11^,^16^,^17^,^18^,^19^. However, TMOs typically suffer from poor inherent electrical and ionic conductivities when applied in electrochemical devices such as lithium ion batteries (LIBs) and electro-chemical capacitors (ECs). To address the inherent limitations of TMOs, exciting progress has recently been made in hybridizing TMOs with a conductive matrix^5^,^20^,^21^,^22^,^23^,^24^. In particular, hierarchical assembly of TMO nanosheets (NSs) on a framework of CNTs (denoted as TMO@CNT composites) has drawn considerable attention^12^,^16^,^25^,^26^. In industry, the design of a hollow structure inside the nanotube facilitates the conduction of heat, while the lamellar structure outside maximizes the surface area and increases the cooling efficiency^27^,^28^. Similarly the design of nanosheet sandwich structures with an interior network of carbon nanotubes can enhance the electron transport from within while the outside layer can facilitate ion transport which could bring benefits to applications in various electro-chemical technologies^13^,^16^,^25^,^29^,^30^,^31^,^32^.

Despite the great potential of these TMO@CNT composites, a general methodology for their controllable synthesis is not yet well established, and it still remains a great challenge to construct high-quality TMO@CNT composite materials in a facile manner. Two characteristics of CNT are considered as major obstacles: they exhibit a strong tendency to agglomerate in order to minimize the total surface free energy and a lack of abundant functional surface groups results in poor compatibility with TMO components^33^,^34^,^35^,^36^. Therefore, appropriate surface functionalization is a critical step in the production of delicate hybrid nanostructures. Conventional surface modifications can introduce hydrophilic groups such as –COOH and –OH albeit at the cost of breaking the pristine graphitic CNT structure. Alternative modification approaches for easy growth and/or deposition of inorganic components are highly demanded and the focus of considerable research attention. For example, Du *et al*. attempted to use layer-by-layer assembly to assist the growth of metal oxide layers on CNT, which requires tedious procedures to functionalize the surface of CNT^37^ while Yang and coworkers adopted gel polymer inducers to assist the preparation of nanostructured inorganic/polymer composites^38^,^39^,^40^. Previously, we have also applied gel polymers to induce the growth of metal oxide nanosheets to fabricate hollow-structured materials^41^,^42^. Despite the great progress to date, a universal method to functionalize the surface of CNT and to simultaneously control the coaxial growth of TMO components with morphology control is still absent.

In this work, we have taken inspiration from nature to create hybrid TMO-CNT structures in a simple way with transition metal oxide nanosheets along a carbon nanotube backbone. These structures draw inspiration from lichen and mosses observed growing on bare rocks and bricks. We noticed the growth of lichen position, and found an intermediate layer between the stones and mosses plays an important role for various types of lichen growth (Figure S1). The key of our strategy relies on the pre-coating of a gel polymer layer of sulfonated-polystyrene on the CNT which induces the facile and uniform growth of various TMO nanosheets. Finally, this general synthetic route realized controllable synthesis towards hierarchical hybrid structures composed of CNT backbones and various species of TMO nanosheets, including NiO, Co_3_O_4_, NiCoO_2_ and ZnCo_2_O_4_. Owing to the intimate contact between CNT and TMOs and their synergistic effect, the charge transfer process in the hybrid nanostructures is significantly enhanced. As a result, these TMO@CNT composite materials demonstrate excellent electrochemical properties when used as electrode materials in lithium ion batteries and electrochemical capacitors. According to previous reports^20^,^21^,^22^, acid treatment was widely used to generate functional groups on the surfaces of CNT, usually via heating CNT in sulfuric/nitric acid at reflux, leading to a large amount of carboxylate ions (–COO–) on the CNT surfaces with negative charge. However, this strategy results in poor morphology control on the as-grown metal oxide nanostructures with unsatisfied coverage of CNT. Herein, we have developed a general strategy for coaxial growth of various transition metal oxide (TMO) nanostructures on CNT by pre-coating a sulfonated polystyrene (SPS). The scheme is illustrated in Fig. 1. CNT with −OH groups (denoted as CNT-OH) were first treated with maleic anhydride in order to introduce vinylic groups onto the CNT surface (Figure S2), which serves as a coupling agent facilitating the *in situ* polymerization of styrene. Thus a polystyrene (PS) layer was coaxially grown on CNT and further treated with H_2_SO_4_, to form a sulfonated PS layer coupled with sulfonic groups (−SO_3_^−^). The CNT-SPS with negative charge can absorb various positively-charged metal ions (eg: Ni^2+^, Co^2+^, Zn^2+^) through mutual electrostatic interactions, followed by mineralization and oxidization during the wet-chemical reaction process and then subsequently calcination. After calcination in nitrogen, delicate TMO@CNT hybrid materials with TMO nanosheets coaxially standing on CNT are produced.

## Materials and Methods

### Materials

Hydroxyle-group functionalized CNT (denote as CNT-OH, Chengdu Organic Chemicals Co. Ltd), Maleic anhydride, azobisisobutyronitrile (AIBN), methylbenzene, acetone, styrene, sulfuric acid, Ni(NO_3_)_2_·6H_2_O, Zn(NO_3_)_2_·6H_2_O, Co(NO_3_)_2_·6H_2_O, hexamethylenetetramine and citric acid trisodium salt dehydrate were all obtained by Aladdin Ltd. (China) were used as received without further treatment.

#### Functionalization of CNT with sulfonated polystyrene (PS@CNT)

For the growth of transition metal oxide nanosheets on CNT, the as-received CNT-OH was firstly functionalized with sulfonated polystyrene, and the functionalization process is illustrated in Figure S2. Firstly, 0.4 g CNT-OH and 8.25 g maleic anhydride were dispersed in 60 mL acetone by sonication in a round-bottom flask. After stirring for 12 hours at room temperature, the centrifugation black precipitate was washed with dry acetone for several times to remove the organic residue. After dried at 60 °C for 10 hours, 0.127 g of the resultant black product above was re-dispersed in 30 mL anhydrous toluene into a three-necked round-bottom flask. 80 mg AIBN and 4.2 mL styrene mixed solution slowly added into the above suspension and then stirred for 3 h at 70 °C under a nitrogen flow. The cooling down the solution to room temperature naturally. The black precipitate, is the PS@CNT, is collected by centrifugation and washed with ethanol several times. The as-prepared PS@CNT were dried in vacuum at 60 °C overnight for use.

#### Sulfonation of CNT-PS (CNT-SPS)

3 g CNT-PS was dispersed into a concentrated sulfuric acid (CNT-PS: H_2_SO_4_ = 1:30, w/w) by sonication, followed by stirring at 40 °C for another 10 min. The sulfonated CNT-PS were obtained by filtering the black precipitate after successively washing with deionized water and ethanol to neutral pH.

#### Synthesis of transition metal (Ni, Co, Zn) oxide nanosheets on CNT (TMO@CNT)

For the synthesis of NiO@CNT, 15 mg CNT-SPS was firstly dispersed in 40 mL deionized water by sonication. 0.5 mmol Ni(NO_3_)_2_·6H_2_O, 0.25 mmol hexamethylenetetramine and 0.025 mmol citric acid trisodium salt dehydrate were added in above suspension. Then, the mixed solution was heated to 90 °C for 6 h. The product of Ni-precursor@CNT was collected by centrifugation, washed with ethanol several times, and dried at 60 °C for 12 h in vacuum. The NiO@CNT was finally obtained by annealing the Ni-precursor@CNT at 400 °C for 2 h under nitrogen atmosphere with a heating ramp rate of 1 °C min^−1^. For the synthesis of Co_3_O_4_@CNT, 0.5 mmol Ni(NO_3_)_2_·6H_2_O was simply replaced with 0.5 mmol Co(NO_3_)_2_·6H_2_O, keeping all other parameters constant, while for the syntheses of NiCoO_2_@CNT and ZnCo_2_O_4_@CNT, 0.25 mmol Ni(NO_3_)_2_·6H_2_O and 0.25 mmol Co(NO_3_)_2_·6H_2_O, and 0.25 mmol Zn(NO_3_)_2_·6H_2_O and 0.5 mmol Co(NO_3_)_2_·6H_2_O were used, respectively.

### Characterization

FESEM images were obtained by a HITACHI su-8010 microscope and TEM images were obtained by a JEOL JEM-2100 microscope. FT-IR spectrum of the SPS@CNT was characterized by a BRUKER Tensor 27 FT-IR spectrophotometer. The phase structures were examined with SHIMADZU Lab X XRD-6000 X-ray diffractometer. Brunauer-Emmett-Teller (BET) method was applied to measure the specific surface area of the products by nitrogen adsorption–desorption isotherms at 77 K in an Autosorb iQ-MP Surface Area and Pore Size Analyzer (Quantachrome Instruments).

#### Electrochemical Measurements

For LIB and EC applications, the working electrodes were prepared by spreading a paste containing the active material (ZnCo_2_O_4_@CNT and NiCoO_2_@CNT), carbon black (super-P-Li) and polymer binder (polyvinylidene difluoride, PVDF, Aldrich) in a weight ratio of 70:20:10.

As electrode for LIBs, 1.1 mg as-prepared ZnCo_2_O_4_ NSs@CNT paste was spread onto a copper foil and then dried at 120 °C overnight in a vacuum oven, followed by pressing at 10 MPa. 1.0 M LiPF_6_ in ethylene carbonate (EC) and diethyl carbonate (DMC) (50:50, w/w) used as the electrolyte. CR2025 coin cells assembly was carried out in an Ar-filled glovebox. CV tests were performed on an electrochemical workstation (CHI 660E, Chenhua, Shanghai). Galvanostatic charge-discharge measurements were carried out using a battery testing system (NEWARE, Shenzhen).

As electrode for ECs, 2 mg NiCo_2_O_4_ slurry was spread onto a graphite paper of 2 cm^2^ and then dried at 120 °C overnight under vacuum. The electrochemical test was conducted with a three-electrode cell in a CHI 660E electrochemical workstation. A Pt electrode as counter electrode, a saturated calomel electrode (SCE) used as the reference electrode, and 2.0 M KOH aqueous solution was used as electrolyte.

The specific capacities were reported based on the amount of the active material, including both TMO nanosheets and CNT, not including the weight of the additives in the electrode.

## Results and Discussion

**Figure 2:**
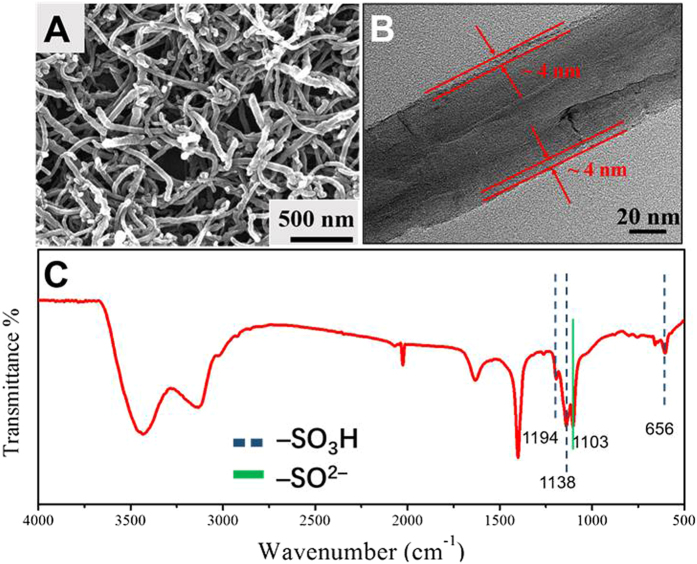
(**A**) SEM image, (**B**) TEM image and (**C**) FT-IR spectrum of SPS-CNT.

The morphological structure of the CNT coated with a layer of sulfonated polystyrene (SPS-CNT) was examined by scanning electron microscopy (SEM) and transmission electron microscopy (TEM). Figure 2A shows that SPS-CNT displays are randomly aligned with lengths of ~2–5 μm. The TEM image in Fig. 2B shows a single carbon nanotube with a diameter of ~120 nm, on which a very thin layer of SPS with thickness of ~4 nm can be clearly observed. The FT-IR spectrum apparently reveals the presence of SPS layer on the CNT (Fig. 2C). The characteristic bands at 1194, 1138 and 656 cm^−1^ can be assigned to the sulfonic acid group (−SO_3_H). The band at 1103 cm^−1^ is related to the sulfone group (−SO_2_^−^). Compared with previous reports^43^, all the characteristic bands are slightly red-shifted due to the conjugation effect between SPS layer and CNT backbone.

**Figure 3:**
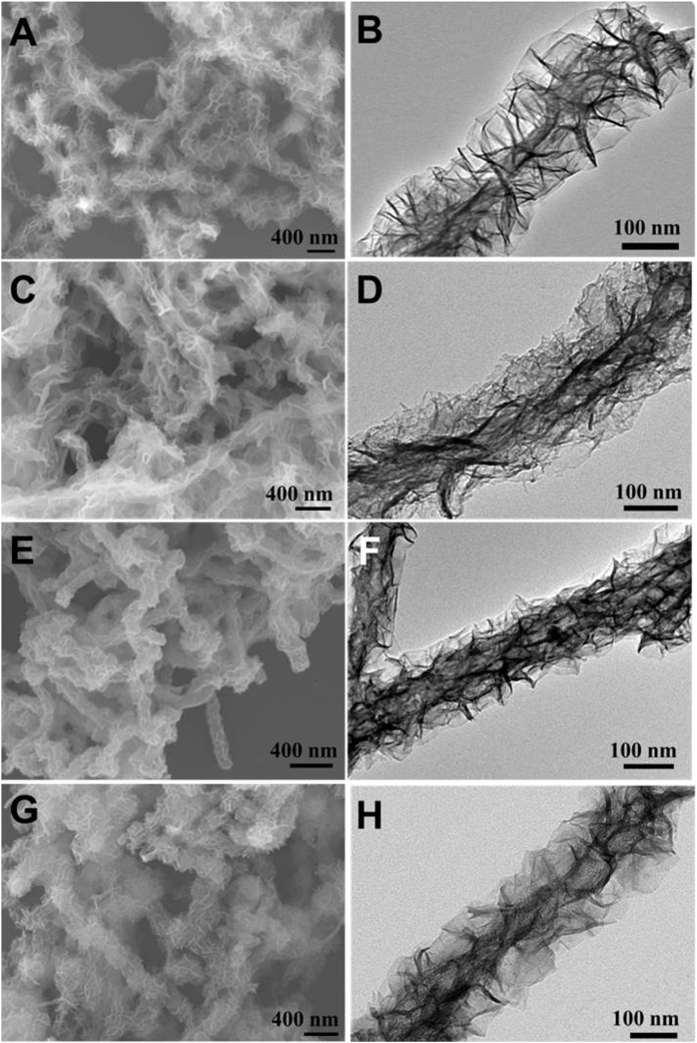
SEM and TEM images of (**A**,**B**) Ni-precursor@CNT, (**C**,**D**) Co-precursor@CNT, (**E**,**F**) NiCo-precursor@CNT and (**G**,**H**) ZnCo-precursor@CNT.

After functionalizing CNT with a SPS layer, various sheet-like metal-containing precursors can be easily grown on CNT via a facile solution-based method. The procedure is illustrated in Fig. 3. Even though different transition metal ions such as unitary Ni^2+^, Co^2+^ and binary Ni^2+^-Co^2+^ and Zn^2+^-Co^2+^ were used, Ni-, Co-, NiCo-, and ZnCo-precursor@CNT-SPS composites were successfully prepared with transition metal precursors coaxially grown on the one-dimensional (1D) CNT with increased diameters up to ~150 nm (Fig. 3A,C,E and G). The TEM images taken from single 1D hybrid nanostructures clearly reveal that ultrathin nanosheets stand on the CNTs, despite the slight difference in the size and growth density (Fig. 3B,D,F and H). Such hierarchical nanostructures with nanosheet shells uniformly and coaxially standing on the CNT cores exhibits porous structure with large surface area (Figure S3, Table S1), which may greatly facilitate the exotic chemical transport and interfacial reaction.

To verify the important role of CNT and their functionalization with SPS in the formation of the unique 1D hierarchical composite structure, additional experiments were carried out without the presence of CNT or with pristine CNT (CNT-OH) during the syntheses. When no CNT were added into the reaction systems, only spherical aggregates with mirco-scale size and poor uniformity were obtained, which were composed of densely stacked sheet-like subunits (Figure S4: A, C, E and G). On the other hand, if CNT functionalized with −OH but without SPS layer were introduced for the synthesis, mixtures of CNT and irregularly assembled sheet-like particles were obtained (Figure S4: B, D, F and H). These results demonstrate that the SPS coating on CNT is critical for the successful and coaxial growth of transition metal derivatives on CNT.

**Figure 4:**
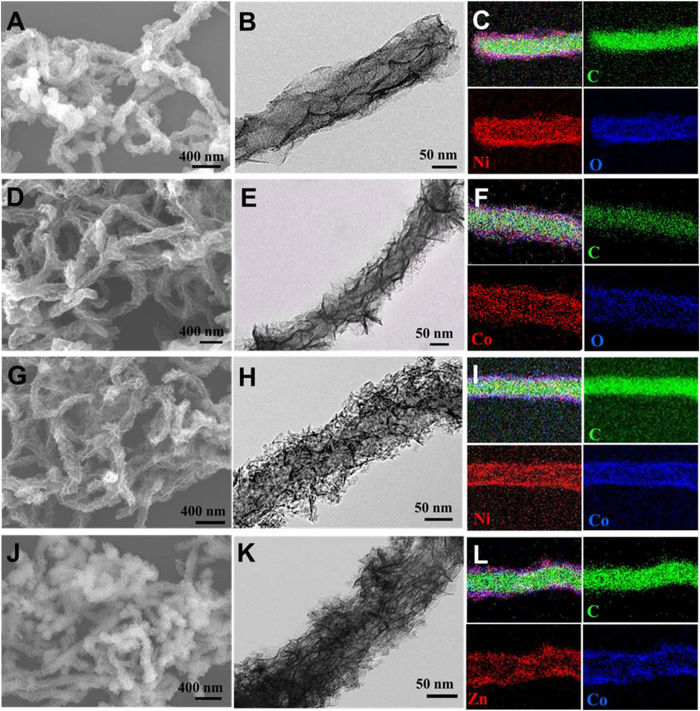
SEM, TEM and EDS mapping images of (**A**,**B** and **C**) NiO@CNT, (**D**,**E** and **F**) Co_3_O_4_@CNT, (**G**,**H** and **I**) NiCoO_2_@CNT and (**J**,**K** and **L**) ZnCo_2_O_4_@CNT.

The metal-precursors on CNT were confirmed to be metal hydroxide (Figure S5), which needed further annealing in inert gas in order to convert into corresponding metal oxides and maintain their composite structure with CNT. Figure 4 shows the morphologies and microstructures of TMO@CNT composites after annealing at 400 °C for 2 hours under nitrogen flow. It can be observed that the overall morphologies, especially the nanosheet structures of the different TMO@CNT composites are well preserved after annealing, suggesting the excellent thermal stability of the samples (Fig. 4A,D,G and J). Meanwhile, the surfaces become coarse and porous (Fig. 4B,E,H and K) after the thermal decomposition of the TMO-precursors and subsequent recrystallization process. Table S1 compares the BET specific surface areas of the TMO-precursor@CNT and the as-obtained TMO@CNT composites, showing that annealing further enlarged the surface area of these materials with improved chemical stability and activity. The elemental composition and distribution of the TMO@CNT composites were further analyzed by energy-dispersive X-ray spectroscopy (EDS) in scanning transmission electron microscopy (STEM), as shown ordinally in Fig. 4C,F,I and H. The EDS mappings clearly show the different spatial distributions of the two components, namely carbon corresponding to CNT core and metal oxides corresponding to the TMO shell. Carbon is located towards the center region of the hybrid structure and metal oxides mainly cover the outer region, further confirming the uniform core-shell structure.

The crystal phases of the samples were characterized by X-ray diffraction (XRD) (Figure S6). All the samples show a peak at 2θ = 26°, corresponding to the (002) diffraction of CNT^9^. In addition, the peak intensity of CNT is less distinguished, suggesting the good coverage of CNT by TMOs and the relatively low CNT content in the composite. Moreover, the metal precursors on CNT were converted into their corresponding oxides. Figure S6A shows that all the other peaks can be indexed to the monoclinic NiO (JCPDS: 47-1049)^44^,^45^, indicating the Ni-precursor@CNT were converted into NiO@CNT after annealing. Similarly, the XRD patterns can be correspondingly assigned to Co_3_O_4_@CNT (JCPDS: 42-1467, Figure S6B)^46^,^47^, NiCoO_2_@CNT (JCPDS: 10-0188, Figure S6C)^48^,^49^ and ZnCo_2_O_4_@CNT (JCPDS: 23-1390, Figure S6D)^50^.

Electrochemical impedance spectroscopy (EIS) was carried out in order to demonstrate the potential electrochemical applications of such TMO@CNT composites. Figure S10 shows the EIS Nyquist plots of pure TMOs and TMO@CNT composites, in which the charge-transfer resistance of the electrode can be revealed by the semi-circle diameter at the high frequency region. Apparently, all TMO@CNT composites exhibit much smaller electron transfer resistance than that of corresponding pure TMOs, indicating the fast electron transfer after the introduction of electrically conductive CNTs. The ternary oxides of NiCoO_2_ and ZnCo_2_O_4_ display a smaller semicircle diameter than that of the binary oxides of NiO and Co_3_O_4_, indicating the improved electron-transfer performance can be achieved by composition engineering of mixed metal oxides, which is consistent with previous reports^50^,^51^.

**Figure 5:**
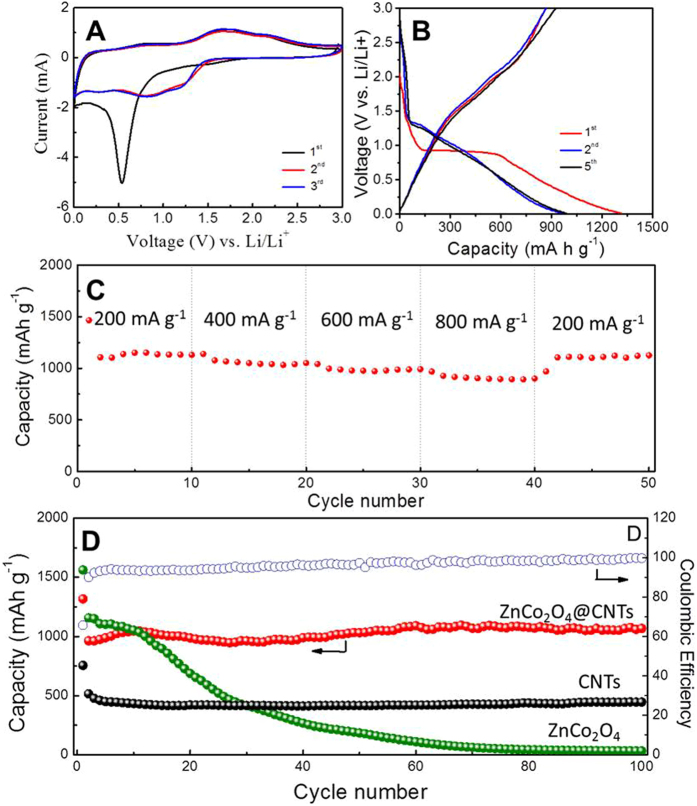
(**A**) CV curves of the ZnCo_2_O_4_@CNT electrode at a scan rate of 0.5 mVs^−1^ between 0.01 V and 3.0 V. (**B**) Charge-discharge voltage profiles of the ZnCo_2_O_4_@CNT electrode at a current density of 400 mAg^−1^. (**C**) Rate capability of ZnCo_2_O_4_@CNT electrode at different current densities. (**D**) Comparison of cycling performance of ZnCo_2_O_4_@CNT (curve I), CNT (curve II) and pure ZnCo_2_O_4_ (curve III) at a current density of 400 mAg^−1^. Curve IV shows the corresponding coulombic efficiency of the ZnCo_2_O_4_@CNT electrode.

The as-prepared TMO@CNT composites were examined as electrode materials for LIBs. Among these binary oxides (NiO and Co_3_O_4_) and ternary oxides (NiCoO_2_, ZnCo_2_O_4_), ZnCo_2_O_4_ with a higher theoretical capacity^50^,^51^,^52^ demonstrated the best lithium storage properties when anchoring on the CNT as electrode. Figure 5A depicts the cyclic voltammograms (CVs) of the electrode made from the ZnCo_2_O_4_@CNT for the initial three cycles at a scan rate of 0.5 mVs^−1^. In the first cycle, the intense peak located at ~0.53 V can be assigned to the reduction of Co^3+^ and Zn^2+^ to metallic Co and Zn, as well as the formation of a solid electrolyte interface (SEI)^14^,^50^,^51^. In the following anodic scan, there are two oxidation peaks appeared at ~1.7 V and ~2.1 V, which can be attributed to the oxidation process of metallic Zn and Co, respectively. The charge and discharge processes can be described as follows^50^,^51^,^53^,^54^,^55^:


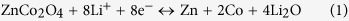



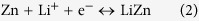



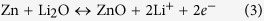



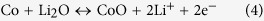



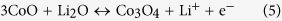


Figure 5B presents the charge-discharge voltage profiles of ZnCo_2_O_4_@CNT for the 1^st^, 2^nd^ and 5^th^ cycles. Coinciding with the CV analysis above, there are two slanted platform with gradually decreasing voltage appeared. The initial discharge and charge capacities are 1317 mAh g^−1^ and 865 mAh g^−1^ at 200 mAg^−1^, respectively. The irreversible capacity loss during the first cycle is about 452 mAh g^−1^, which can be attributed to the incomplete decomposition of a SEI film^56^,^57^,^58^,^59^.

Figure 5C shows that the ZnCo_2_O_4_@CNT electrode exhibits excellent rate capability at different current densities. Benefitted from the unique structure, even at a high current density of 800 mAg^−1^, the ZnCo_2_O_4_@CNT electrode still deliver a discharge capacity of 900 mAh g^−1^. Figure 5D depicts the comparative cycling performance of three different samples: pure commercial CNT, pure ZnCo_2_O_4_ and ZnCo_2_O_4_@CNT at the same current density of 400 mAg^−1^. The ZnCo_2_O_4_@CNT electrode shows an excellent stable cycling performance of 1068 mAh g^−1^ after 100 cycles. Whereas, the pure ZnCo_2_O_4_ electrode shows a rapidly decreasing along with cycling, only retaining 33.8 mAh g^−1^ after the same cycles, while CNT electrode exhibit a lower reversible capacity of 490 mAh g^−1^. Apparently, the ZnCo_2_O_4_@CNT electrode manifests the best cycling performance. The excellent lithium storage properties of ZnCo_2_O_4_@CNT can be attributed to the unique composite structure. On one hand, CNT possess good electrical conductivity, high mechanical strength and flexibility, facilitating the efficient charge transfer and maintaining the structural integrity of the electrode during the repeating charge-discharge processes. On the other hand, the ultrathin ZnCo_2_O_4_ nanosheets coaxially standing on CNT provide numerous active sites for lithium storage due to their large surface area. The combination of the high lithium storage capacity of TMOs and the buffering effect of conductive CNT matrix contributes to an enhanced electrochemical performance^29^. Besides, we also investigate the lithium storage properties of NiO@CNT (Figure S7), Co_3_O_4_@CNT (Figure S8) and NiCoO_2_@CNT (Figure S9) electrodes and prove whole these materials exhibiting superior capacitances and nice stabilities.

**Figure 6:**
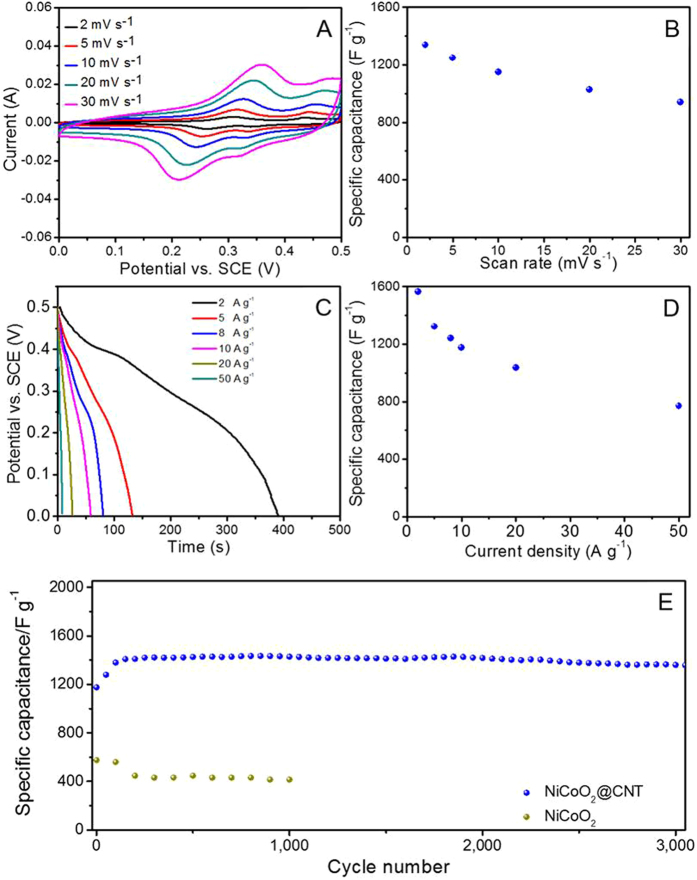
Electrochemical characterization of NiCoO_2_@CNT. (**A**) CV curves at various scan rates ranging from 2 to 30 mVs^−1^. (**B**) average specific capacitance of NiCoO_2_@CNT at various scan rates. (**C**) Discharge voltage profiles at various scan rates ranging from 2 to 50 Ag^−1^. (**D**) The calculated capacitance as a function of current density according to data in (**C**). (**E**) Specific capacitance versus cycle number of NiCoO_2_@CNT at a current density of 10 Ag^−1^.

Electrochemical capacitors are an alternative type of device which can storage and release energy rapidly and reversibly^2^,^3^,^60^. The electrochemical capacitive properties of TMOs and TMO@CNT composites were also investigated and the as-prepared NiCoO_2_@CNT was found to demonstrate the best performance. Figure 6A shows the CV curves of the NiCoO_2_@CNT electrode at scan rates from 2 to 50 mVs^−1^. The shape of the CV curves reveals the typical pseudocapacitive characteristics of the sample. One pair of redox peaks within the potential range from 0 to 0.5 V vs. a saturated calomel electrode (SCE). It can be attributed to the reversible reduction as described by reactions (6) and (7) below^61^:


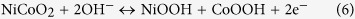



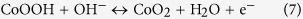


Along with the rate increasing, except for a little shift of the peaks position, the shape of CV curves shows no significant change, thus indicating great electrochemical reversibility and excellent rate performance. As shown in Fig. 6B, the average capacitances of NiCoO_2_@CNT are calculated to be 1335, 1248, 1150, 1025 and 939 Fg^−1^ at scan rates of 2, 5, 10, 20 and 30 mVs^−1^, respectively. Figure 6C shows the galvanostatic discharge curves of the NiCoO_2_@CNT at different current densities ranging from 2 to 50 Ag^−1^. There are two clear plateaus appearing in each discharge curve, which are consistent with the CV analysis. Each specific capacitance at different rate can be calculated via equation (8):


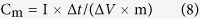


where C_m_ (F g^−1^) is the specific capacitance, I (A) is the discharge current, Δt (s) is the discharge time, ΔV is the potential change during the discharge process, and the m (g) is the mass of the active materials (NiCoO_2_@CNT) in the electrode. The calculated specific capacitance at different discharge current density shown in Fig. 6D, giving high specific capacitances of 1564, 1322, 1240, 1176, and 1036 Fg^−1^ at different current densities of 2, 5, 8, 10, 20 and 50 Ag^−1^, respectively. Figure 6E display the specific capacitance at a current density of 10 Ag^−1^ of 3000 cycles for investigating the performance of cycling stability. The specific capacitance is around 1176 Fg^−1^ in the first cycle, and it slightly increases to 1380 Fg^−1^ in the course of first 100 cycles, which still keeps a capacitance of 1360 Fg^−1^ after 3000 cycles with an overall capacitance loss of only 1.4%. The excellent electrochemical performance further highlights the capability of the NiCoO_2_@CNT composite electrode to meet the requirements of both long cycling performance and good rate capability, which are promising for practical application as energy storage devices.

In summary, we have developed a general strategy to synthesize various TMO@CNT composite materials with the assistance of pre-coated sulfonated-polystyrene (SPS) layers on CNT. The SPS layer effectively facilitate the growth of metal-precursor nanosheets under wet-chemical synthesis, which can be conformably converted into metal oxide nanosheets by annealing under inert atmosphere, leading to the formation of core-shell hybrid hierarchical nanostructures. Owing to the unique hybrid nanostructure and strong coupling effect between CNT and TMOs, improved charge transfer process and large active surface area are achieved, which results in excellent electrochemical lithium/charge storage properties when evaluated as electrode materials for lithium ion batteries and electrochemical capacitors. More importantly, the general synthetic approach allows the readily engineering of compositions of the shell components with delicate morphologies on CNT, which would be greatly promising to explore advanced functional materials with potential applications in various emerging technologies, such as energy storage/conversion systems, electronic devices, and sensors.

## Additional Information

**How to cite this article**: Zhou, H. *et al*. A universal synthetic route to carbon nanotube/transition metal oxide nano-composites for lithium ion batteries and electrochemical capacitors. *Sci. Rep.* **6**, 37752; doi: 10.1038/srep37752 (2016).

**Publisher's note:** Springer Nature remains neutral with regard to jurisdictional claims in published maps and institutional affiliations.

## Supplementary Material

Supplementary Information

## Figures and Tables

**Figure 1:**
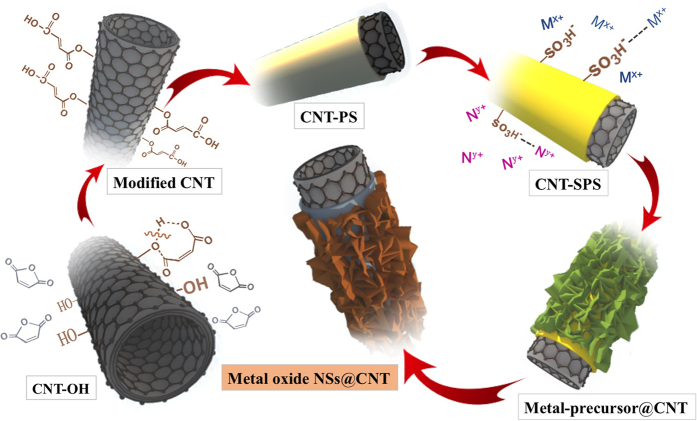
Schematic illustration of the synthetic procedure for TMO@CNT hybrid materials through pre-coating CNT with sulfonated polystyrene.
