# Supplementary material for: A universal synthetic route to carbon nanotube/transition metal oxide nano-composites for lithium ion batteries and electrochemical capacitors
Source: Sci Rep. 2016 Nov 25;6:37752. doi: 10.1038/srep37752 (PMC5123580; doi:10.1038/srep37752)
Supplement: Supplementary Information [file srep37752-s1.doc]

**Supporting information**

**A universal synthetic route to carbon nanotube/transition metal oxide nano-composites for lithium ion batteries and electrochemical capacitors**

*Han Zhou ^a#^, Lusi Zhang ^a#^, Dongyang Zhang^a^, Shuangqiang Chen ^c^, Paul R. Coxon ^b^, Xiong He ^b^, Mike Coto ^b^, Hyun-Kyung Kim ^b^, Kai Xi ^b*^ and Shujiang Ding ^a*^*

^a^Department of Applied Chemistry, School of Science, MOE Key Laboratory for Nonequilibrium Synthesis and Modulation of Condensed Matter, State Key Laboratory for Mechanical Behaviour of Materials, Xi’an Jiaotong University, Xi’an China

^b^Department of Materials Science and Metallurgy, University of Cambridge, Cambridge CB3 0FS, United Kingdom

^c^Centre for Clean Energy Technology, School of Mathematical and Physical Sciences, University of Technology Sydney, 15 Broadway, Sydney, New South Wales, 2007, Australia

^*^Corresponding author: kx210@cam.ac.uk

dingsj@xjtu.edu.cn

^#^H. Zhou and L. Zhang equally contributed to this work.


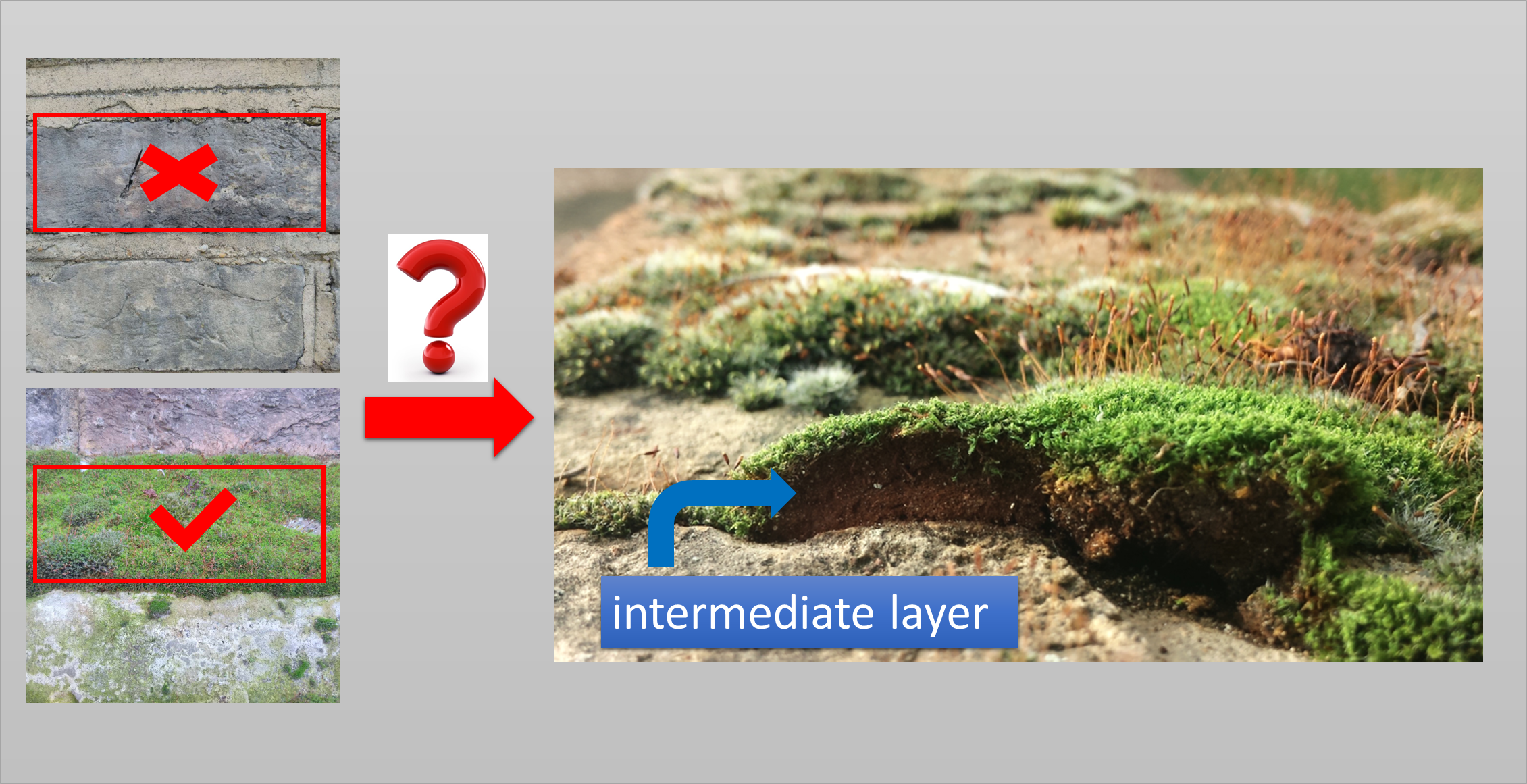


**Figure S1**. Lichen and mosses are growing on the exterior walls of Jesus College, Cambridge. An intermediate layer between the stones and mosses play an important role for various types of lichen growth.


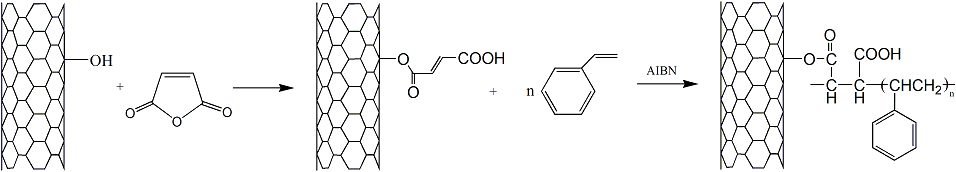


**Figure S2**. Illustration of the synthetic procedure of CNT-PS.


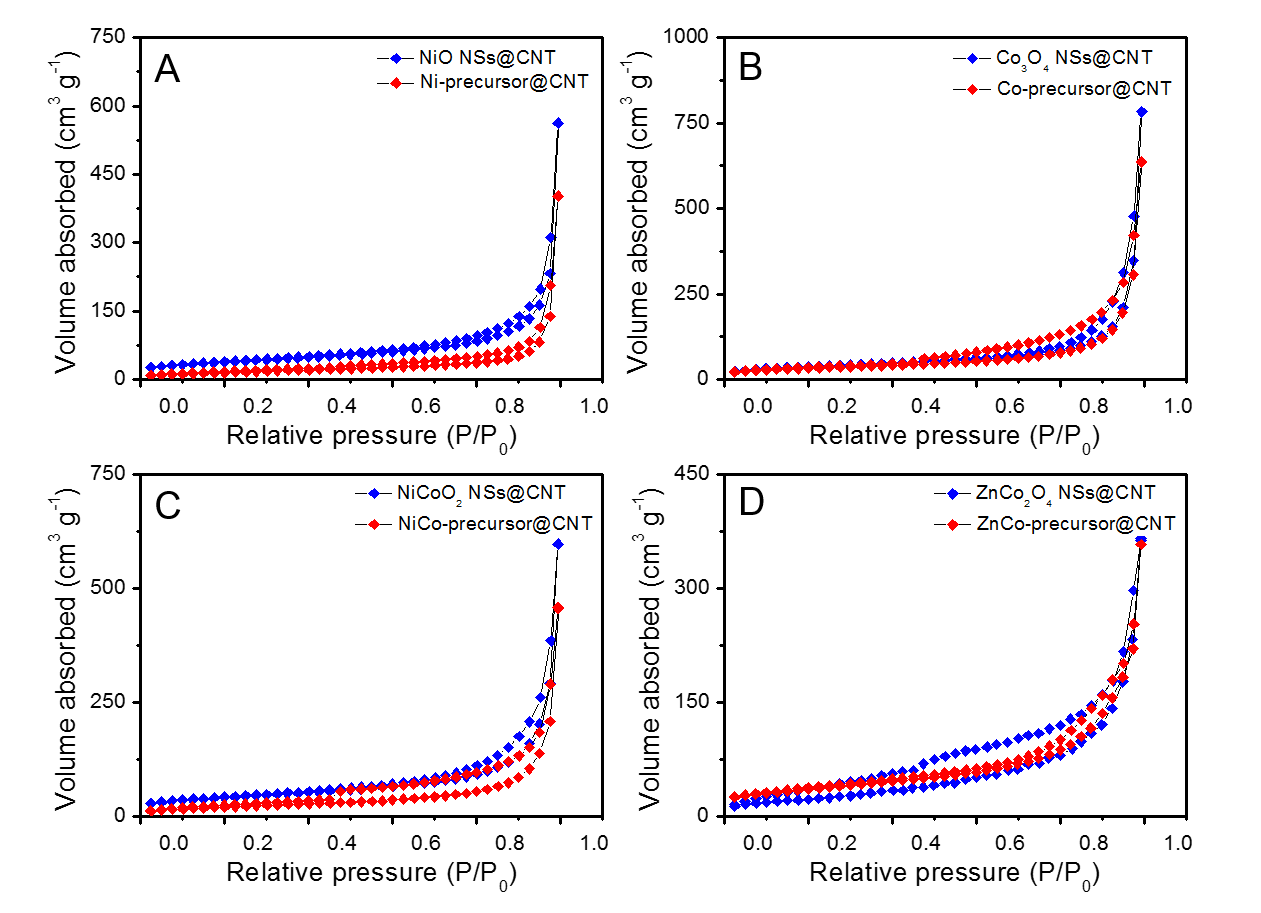


**Figure S3**. N_2_ adsorption–desorption isotherms of A) NiO NSs@CNT and Ni-precursor@CNT; B) Co_3_O_4_ NSs@CNT and Co-precursor@CNT; C) NiCoO_2_ NSs@CNT and NiCo-precursor@CNT and D) ZnCo_2_O_4_ NSs@CNT and ZnCo-precursor@CNT.

**Table S1**. Surface Areas of Ni-precursor@CNT, NiO@CNT, Co-precursor@CNT, Co_3_O_4_@CNT, NiCo-precursor@CNT, NiCoO_2_@CNT, ZnCo-precursor@CNT and ZnCo_2_O_4_@CNT.

| Materials | Surface Area (m^2^ g^-1^) | Materials | Surface Area (m^2^ g^-1^) |
| --- | --- | --- | --- |
| Ni-precursor@CNT | 97.981 | NiO@CNT | 136.012 |
| Co-precursor@CNT | 115.753 | Co_3_O_4_@CNT | 127.409 |
| NiCo-precursor@CNT | 154.089 | NiCoO_2_@CNT | 160.045 |
| ZnCo-precursor@CNT | 85.9 | ZnCo_2_O_4_@CNT | 127.942 |


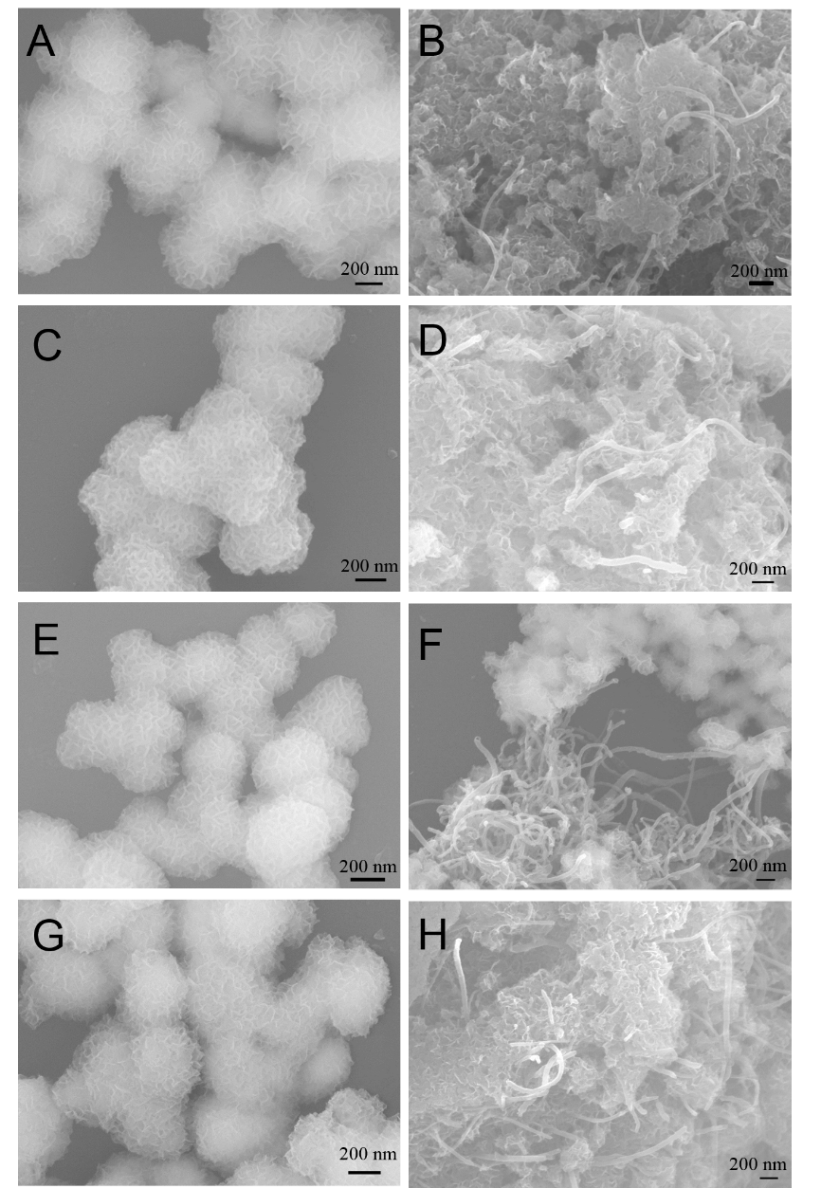


**Figure S4**. SEM images of A) Ni-precursor particles without CNT; B) Niprecursor prepared with CNT-OH; C) Co-precursor particles without CNT; D) Co-precursor prepared with CNT-OH; E) NiCo-precursor particles without CNT; F) NiCo-precursor prepared with CNT-OH; G) ZnCo-precursor particles without CNT; H) ZnCo-precursor prepared with CNT-OH.


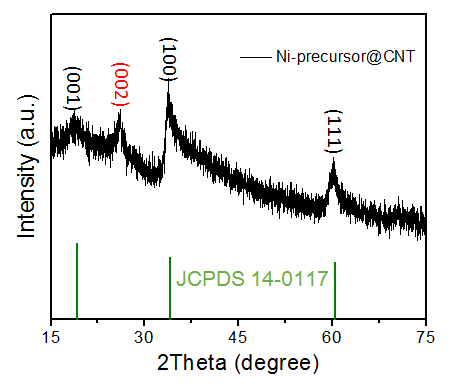


**Figure S5**. XRD pattern of the Ni-precursor@CNT, which can be indexed to the β-Ni(OH)_2_ phase (JCPDS No. 14-0117).


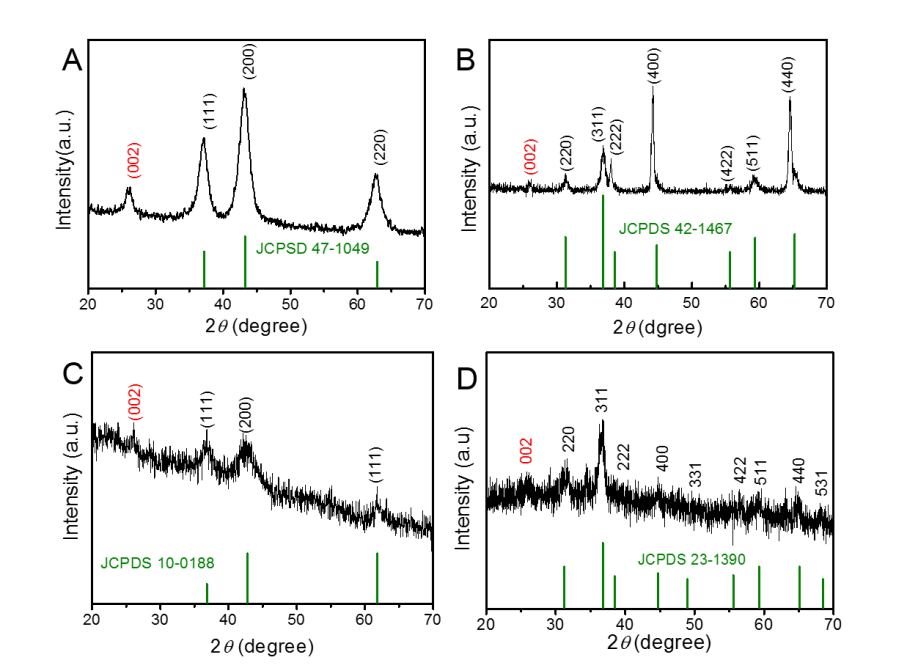


**Figure S6**. XRD patterns of (A) NiO@CNT, (B) Co_3_O_4_@CNT, (C) NiCoO_2_@CNT and (D) ZnCo_2_O_4_@CNT.


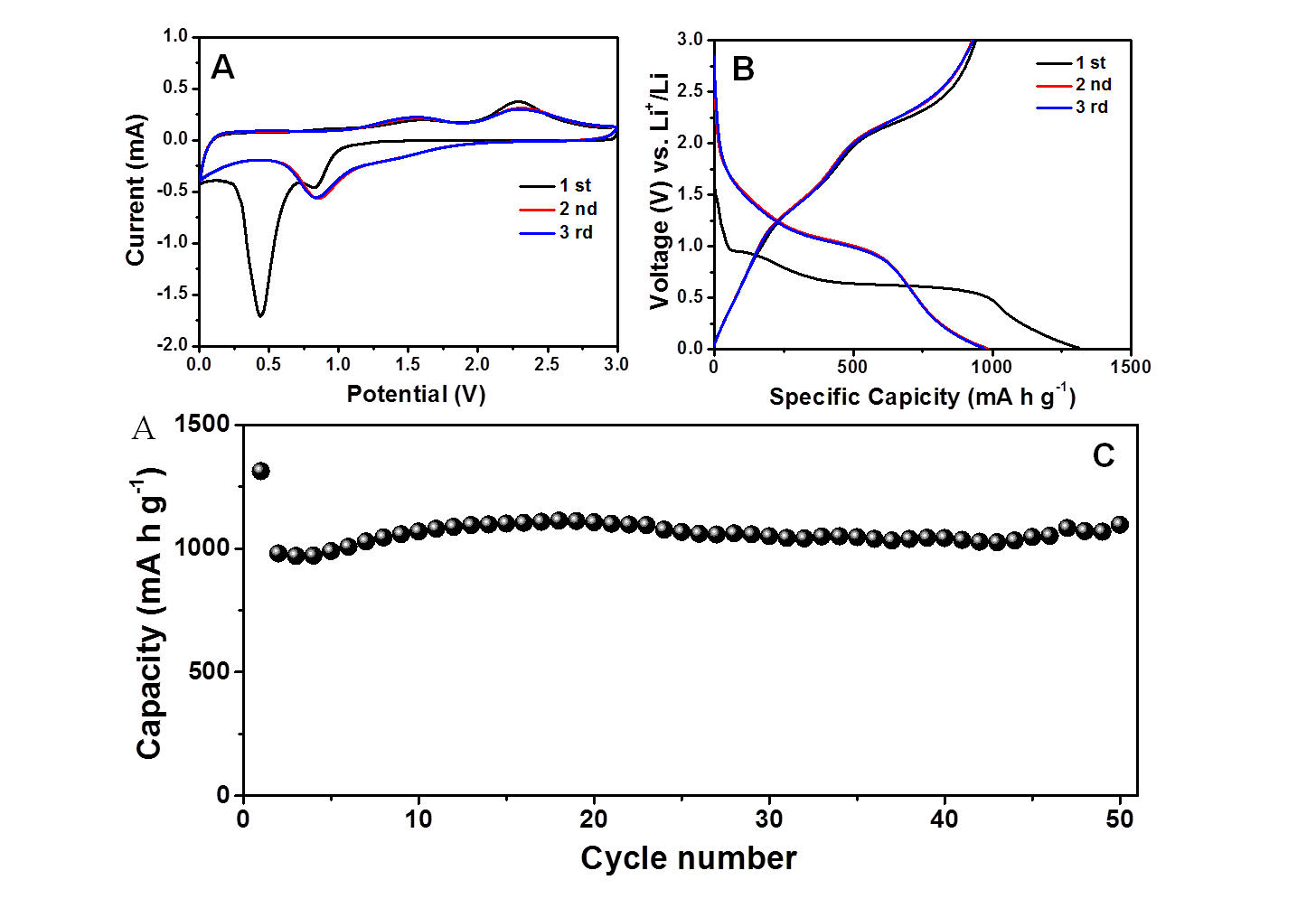


**Figure S7.** (A) CV curves of the NiO@CNT electrode at a scan rate of 0.5 mV s^-1^ between 0.01 V and 3.0 V. (B) Charge-discharge voltage profiles of the NiO@CNT electrode at a current density of 400 mA g^-1^. (C) Cycling performance of NiO@CNT current density of 400 mA g^-1^.


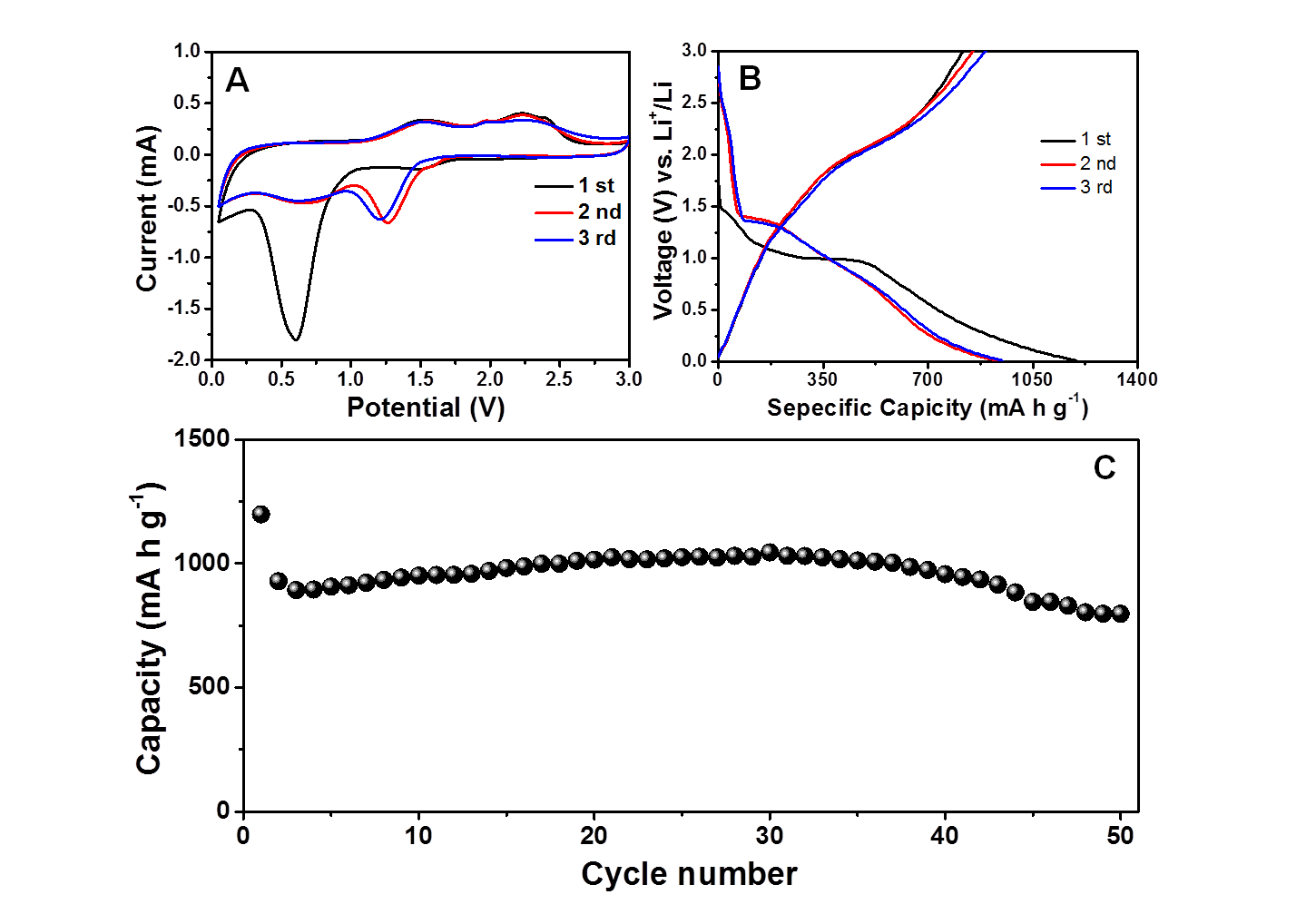


**Figure S8**. (A) CV curves of the Co_3_O_4_@CNT electrode at a scan rate of 0.5 mV s^-1^ between 0.01 V and 3.0 V. (B) Charge-discharge voltage profiles of the Co_3_O_4_@CNT electrode at a current density of 400 mA g^-1^. (C) Cycling performance of Co_3_O_4_@CNT current density of 400 mA g^-1^.


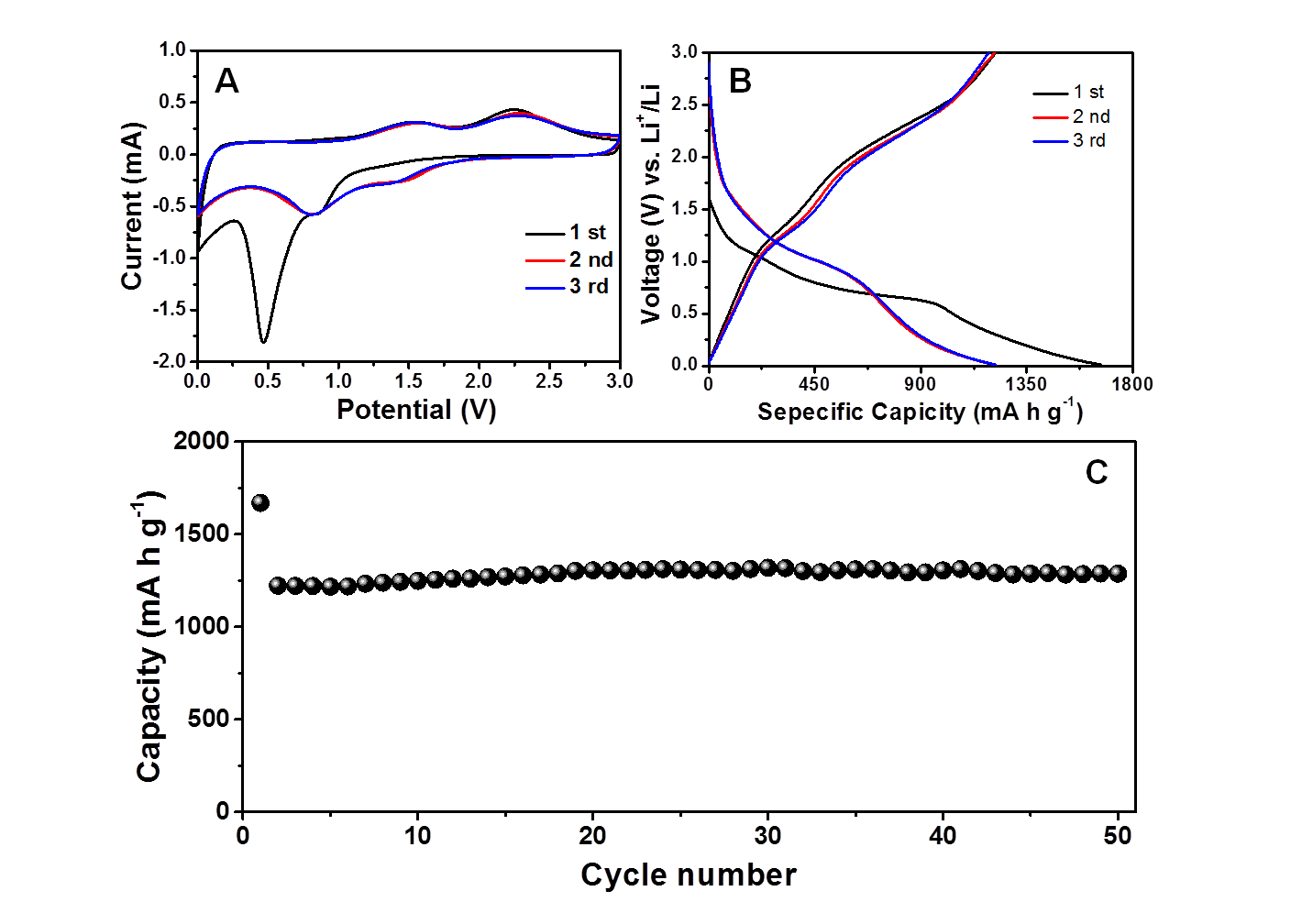


**Figure S9**. (A) CV curves of the NiCoO_2_@CNT electrode at a scan rate of 0.5 mV s^-1^ between 0.01 V and 3.0 V. (B) Charge-discharge voltage profiles of the NiCoO_2_@CNT electrode at a current density of 400 mA g^-1^. (C) Cycling performance of NiCoO_2_@CNT current density of 400 mA g^-1^.


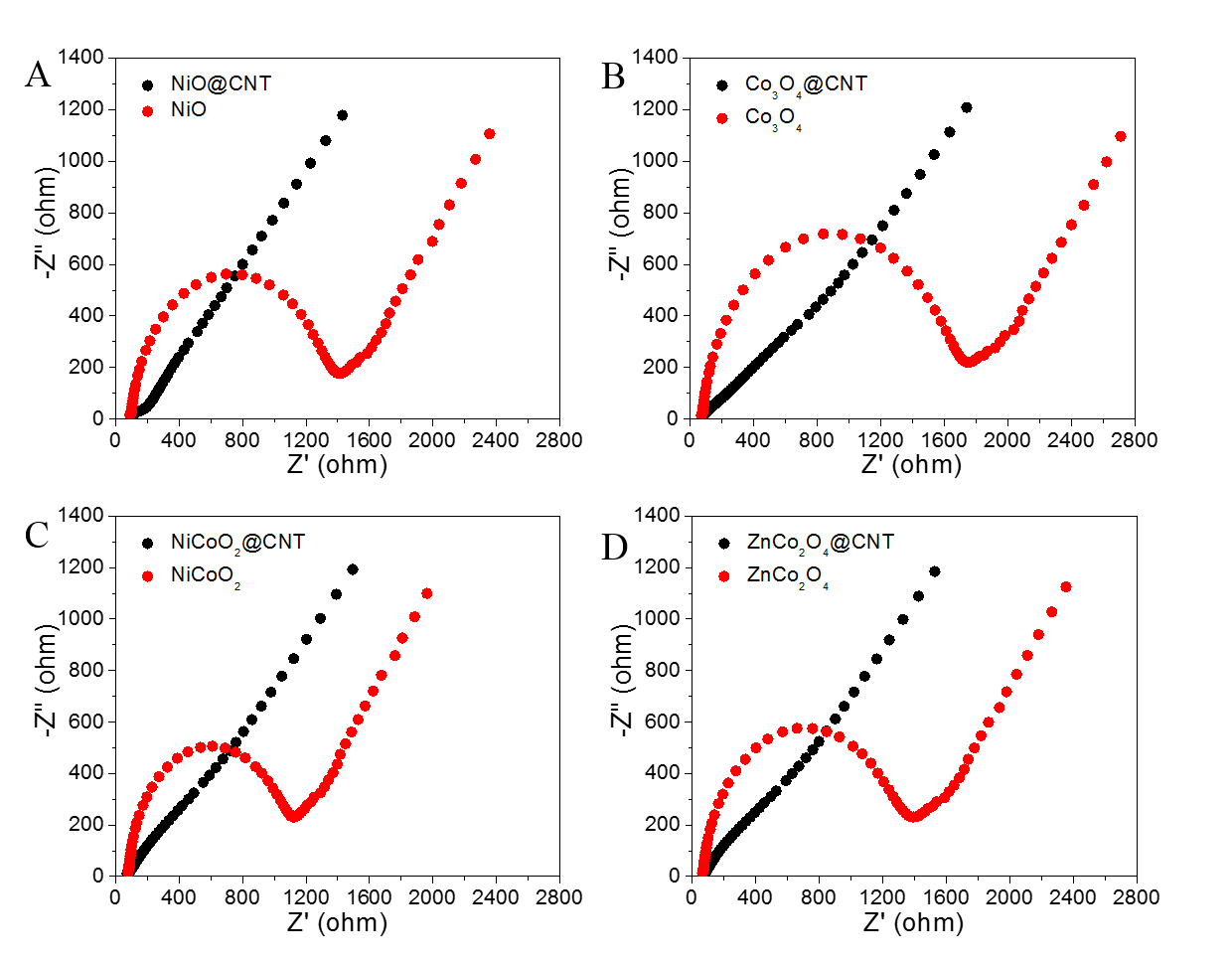


Figure S10. EIS Nyquist plots of (A) NiO@CNT and NiO, (B) Co_3_O_4_@CNT and Co_3_O_4_, (C) NiCoO_2_@CNT and NiCoO_2_, (D) ZnCo_2_O_4_@CNT and ZnCo_2_O_4_.
